# Finerenone in Asian patients with diabetic kidney disease: evaluating the evidence for “better efficacy”

**DOI:** 10.1093/ckj/sfag279

**Published:** 2026-08-20

**Authors:** Siqian Gong, Linong Ji

**Affiliations:** Department of Endocrinology and Metabolism, Peking University People’s Hospital, Beijing, China; Beijing Key Laboratory of Innovative Drug and Device Translation in Endocrine and Metabolic Diseases, Beijing, China; Department of Endocrinology and Metabolism, Peking University People’s Hospital, Beijing, China; Beijing Key Laboratory of Innovative Drug and Device Translation in Endocrine and Metabolic Diseases, Beijing, China; Peking University Diabetes Center, Beijing, China

**Keywords:** Asian population, diabetic kidney disease, finerenone, mineralocorticoid receptor antagonist, type 2 diabetes

## Abstract

Diabetic kidney disease (DKD) has emerged as the primary driver of kidney failure across the Asia-Pacific region. Thus, therapeutic interventions capable of effectively slowing the progression of chronic kidney disease (CKD) and reducing concomitant cardiovascular risk are of critical strategic importance. Finerenone, a novel non-steroidal mineralocorticoid receptor antagonist (nsMRA) approved for the treatment of DKD, exerts cardiorenal protective effects by selectively inhibiting the overactivation of the mineralocorticoid receptor (MR)—a key mediator of pathological inflammatory and fibrotic processes underlying renal and cardiac injury. A consistent finding across major clinical trials, including FIDELIO-DKD, FIGARO-DKD, and the pooled FIDELITY analysis, is the “potentially enhanced” (based on post-hoc subgroup analyses) therapeutic efficacy of finerenone in Asian, predominantly Chinese, patient populations. This review synthesizes emerging evidence indicating a distinct, potentially superior therapeutic response (based on available subgroup analyses) in Asian cohorts compared to global populations, alongside potential genetic and environmental factors contributing to this phenomenon. Additionally, we address the associated safety profile of hyperkalemia in Asian patients. We contend that this risk is manageable through structured monitoring and should not preclude clinicians from utilizing this high-impact therapy. Ultimately, this review positions finerenone as a crucial and effective intervention for Asian patients with high cardiorenal risk, provided that potassium homeostasis is proactively managed.

## INTRODUCTION

Diabetic kidney disease (DKD) is a major and growing healthcare burden across the Asia-Pacific region. Epidemiological data show that several Asian countries are among the world’s leaders in DKD as the main cause of kidney failure. This highlights the urgent need for effective treatment strategies tailored to this population [1].

Finerenone is a new non-steroidal mineralocorticoid receptor antagonist (nsMRA). It has become an important therapy, with clear evidence of cardiorenal benefits in patients with type 2 diabetes (T2D) and chronic kidney disease (CKD) [1, 2]. Its proven efficacy has led to its inclusion in major international and regional clinical practice guidelines, such as those from the Asian Pacific Society of Nephrology (APSN) and Kidney Disease: Improving Global Outcomes (KDIGO) [3, 4].

Finerenone’s efficacy is well-established in global patient groups. But new evidence from post-hoc analyses and meta-analyses suggests its therapeutic effects on key kidney and heart-related outcomes may be stronger in Asian patients. This finding has sparked wide clinical and academic interest in whether ethnic, genetic, or environmental factors can affect the drug’s efficacy and safety.

The main goal of this review is to systematically bring together clinical evidence on finerenone’s efficacy and safety in Asian populations—with a specific focus on Chinese patients. We will also explore potential underlying mechanisms, such as genetic differences, dietary habits, and baseline clinical traits. These factors may explain why treatment responses vary between ethnic groups.

It is important to note at the outset that much of the evidence discussed in this review is derived from post-hoc subgroup analyses of the FIDELIO-DKD, FIGARO-DKD, and pooled FIDELITY trials. By design, post-hoc analyses are hypothesis-generating rather than hypothesis-confirming, and are susceptible to type I error, particularly when multiple subgroups are examined. Notably, the formal statistical tests for treatment-by-ethnicity interaction did not reach significance in the parent randomized controlled trials for most endpoints. The findings summarized below should therefore be regarded as a consistent and biologically plausible signal warranting further dedicated study, rather than definitive proof of a superior therapeutic effect in Asian patients.

For clarity, in this review “Asian” refers to the pooled Asian subgroups enrolled in the FIDELIO-DKD, FIGARO-DKD, FIDELITY, and CONFIDENCE trials (predominantly from China, Japan, and Korea, with CONFIDENCE additionally including participants from India and Taiwan), whereas “Chinese” refers specifically to the Chinese subgroup analyses reported separately for FIDELIO-DKD and FIGARO-DKD. Because the majority of the currently available subgroup evidence derives from Chinese cohorts, findings attributed to “Asian” populations are disproportionately influenced by Chinese data. Asia encompasses populations of highly diverse genetic ancestry, dietary habits, and disease burden, and conclusions drawn here should not be uncritically generalized to all Asian populations.

### INSIGHTS FROM FINERENONE’S ASIAN CLINICAL EVIDENCE

#### Greater relative risk reduction for kidney composite endpoints

Two pivotal randomized controlled trials (RCTs) verifying the long-term cardiorenal protective efficacy and safety of finerenone FIDELIO-DKD trial and FIGARO-DKD trial—have designated the composite kidney endpoint as the primary and secondary endpoint, respectively [5, 6]. This composite outcome includes kidney failure, a sustained estimated glomerular filtration rate (eGFR) decline of ≥40% or death from renal causes.

The FIDELIO-DKD trial focused on patients with high-risk (usually advanced) CKD. In its Chinese subgroup [7], finerenone cut the risk of the primary kidney composite endpoint by 41% [hazard ratio (HR) = 0.59, 95% confidence interval (CI): 0.39–0.88, *P* = 0.009], For comparison, the overall relative risk reduction (RRR) in the global trial was only 18%. Based on an absolute between-group difference of 12.2% after 30 months, the NNT to prevent one primary outcome event was eight (95% CI: 4–84).

The FIGARO-DKD trial included patients across a wider range of CKD stages. In the post-hoc analysis of the Chinese subgroup (*n* = 325) from FIGARO-DKD trial [8], finerenone significantly reduced the risk of the key secondary kidney composite outcome by 52% (HR = 0.48, 95% CI: 0.29–0.79, *p* = 0.0029) compared to placebo. This finding is particularly notable when we compare it with the overall FIGARO-DKD population. In that group, the risk reduction for the same composite outcome was not statistically significant (HR = 0.87, 95% CI: 0.76–1.01) [6]. It should be noted that these Chinese subgroup analyses are based on relatively modest sample sizes (*n* = 372 for FIDELIO-DKD and *n* = 325 for FIGARO-DKD), and the resulting hazard ratio estimates carry wide confidence intervals, indicating considerable statistical imprecision. These findings should therefore be interpreted with appropriate caution.

#### Better effect on greater surrogate marker improvement

The sign of “better Asian efficacy” gets more support from a 2024 meta-analysis by Xu *et al*. [2]. The analysis combined data from seven RCTs involving nearly 15 000 participants. It revealed significant differences in how non-steroidal MRAs worked between Asian and non-Asian subgroups on key surrogate markers: (1) Urine albumin-to-creatinine ratio (UACR) reduction: Asian patients had a significantly greater UACR reduction [weighted mean difference (WMD) −0.59] than non-Asian patients (WMD −0.29). (2) Blood pressure reduction: Asians had a more obvious antihypertensive effect. Their systolic blood pressure (SBP) dropped more (WMD −5.12 mmHg) than that of non-Asians (WMD −3.64 mmHg) Table 1.

**Table 1:** Summary of efficacy signals for finerenone in Asian, Chinese, and comparator populations across subgroup analyses and meta-analysis.

Evidence block	Study/analysis	Population level	Endpoint/parameter	Asian/Chinese estimate	Comparator estimate	Interaction P
Clinical outcomes	FIDELIO-DKD	Chinese subgroup,*n* = 372	Kidney composite endpoint, HR (95% CI)	0.59 (0.39–0.88)	0.82 (0.73–0.93) (Global)	NR
Clinical outcomes	FIDELIO-DKD	Asian subgroup,*n* = 1327	Kidney composite endpoint, HR (95% CI)	0.70 (0.56–0.87)	NR	NR
Clinical outcomes	FIGARO-DKD	Chinese subgroup,*n* = 325	Kidney composite endpoint, HR (95% CI)	0.48 (0.29–0.79)	0.87 (0.76–1.01) (Global)	NR
Clinical outcomes	FIDELITY	Asian subgroup, *n* = 2858	CV composite endpoint, HR (95% CI)	0.90 (0.70–1.15)	0.85 (0.77–0.94)	0.845
Clinical outcomes	FIDELITY	Asian subgroup	Kidney composite ≥57% eGFR decline, HR (95% CI)	0.64 (0.50–0.82)	0.85 (0.71–1.00)	0.049
Clinical outcomes	FIDELITY	Asian subgroup	Kidney composite ≥40% eGFR decline, HR (95% CI)	0.67 (0.56–0.80)	0.93 (0.84–1.04)	<0.001
Surrogate markers	Xu *et al*. meta-analysis	Asian vs. non-Asian	UACR reduction, WMD (95% CI)	−0.59 (−0.73 to −0.45)	−0.29 (−0.32 to −0.27)	NR
Surrogate markers	Xu *et al*. meta-analysis	Asian vs. non-Asian	SBP reduction, WMD (95% CI)	−5.12 (−5.84 to −4.41)	−3.64 (−4.38 to −2.89)	NR
Surrogate markers	FIDELITY	Asian subgroup	eGFR slope, LS mean difference (95% CI)	1.08 (0.53–1.63)	NR	NR
Surrogate markers	FIGARO-DKD	Chinese subgroup	eGFR slope, LS mean difference (95% CI)	2.25 (0.75–3.76)	NR	NR
Surrogate markers	FIDELIO-DKD	Chinese subgroup	UACR reduction at month 4	−26.8%	NR	NR
Surrogate markers	FIGARO-DKD	Chinese subgroup	UACR reduction at month 4	−39%	NR	NR
Surrogate markers	FIDELITY	Asian subgroup	UACR reduction at month 4	−33%	NR	NR

CV, cardiovascular; NR, not reported.

Data-availability note: For FIDELIO-DKD and FIGARO-DKD, trial-level Asian-versus-non-Asian estimates and formal treatment-by-ethnicity interaction *P* values were not reported for several endpoints. Values labelled (Global) represent the overall trial estimate, which includes Asian participants, and should not be interpreted as non-Asian-only estimates. Among the sources summarized here, the FIDELITY Asian subgroup analysis provides the clearest Asian-versus-non-Asian comparison with formal interaction testing.

Interpretation note: The estimates summarized in this table are derived largely from subgroup and post-hoc analyses and should be interpreted as hypothesis-generating unless supported by prespecified interaction testing or prospective confirmatory evidence.

A new post-hoc analysis of the FIDELITY program demonstrated significant efficacy in preserving kidney function and reducing albuminuria in the Asian patient subpopulation [9]. In this analysis, a significantly higher proportion of patients treated with finerenone experienced a clinically meaningful improvement in their albuminuria category, indicating a reversal of kidney damage. Specifically, regression from “high” to “normal” albuminuria occurred in 39.5% of finerenone-treated patients versus 14.8% of placebo-treated patients (HR 3.04, 95% CI: 2.21–4.18); regression from “very high” to “normal” albuminuria occurred in 3.8% versus 1.7% (HR 2.27, 95% CI: 1.28–4.05); and regression from “very high” to “high” albuminuria occurred in 42.3% versus 20.3% (HR 2.69, 95% CI: 2.27–3.19). The estimated cumulative incidence of regression from “high” to “normal” albuminuria at 5 years was 72.2% for finerenone versus 18.8% for placebo. The chronic eGFR slope was also reduced with finerenone compared to placebo, the least-squares (LS) mean annual decline between-group difference was 1.08 ml/min/1.73 m² (95% CI: 0.53–1.63; *P* = 0.0002) in favor of finerenone (−2.60 ml/min/1.73 m² for finerenone vs. −3.67 ml/min/1.73 m² for placebo).

Evidence from clinical practice further supports these trial-based observations. A retrospective real-world study of Chinese patients with microalbuminuria conducted at the Peking University First Hospital [10] reported a significant reduction in albuminuria following finerenone treatment. Over a six-month period, the geometric mean for UACR decreased by a remarkable 48%, from a baseline of 99.67 to 51.86 mg/g. This substantial effect in a real-world setting reinforces the potent albuminuria-lowering effect of finerenone in this population.

#### The safety and tolerability profile in Asian patients

Alongside the efficacy, multiple analyses indicate a potentially different safety profile in Asian patients compared to other populations. The most consistent signal relates to higher reported incidence of hyperkalemia (serum K⁺ >5.5 mmol/l), a known class effect of MRAs.

The meta-analysis by Xu *et al*. found that the incidence of hyperkalemia was significantly higher in Asian patients compared to non-Asian patients [odds ratio (OR) 5.78 vs. OR 2.21, respectively] [2]. In the FIGARO-DKD Chinese subgroup, investigator-reported hyperkalemia was notably high in both the finerenone arm (19.1%) and the placebo arm (18.5%), and these rates were substantially higher than those observed in the overall FIGARO-DKD global population [8].

The CONFIDENCE trial was designed to compare the efficacy of simultaneously initiating finerenone and empagliflozin against either drug as a monotherapy for reducing albuminuria [11]. In the CONFIDENCE Asian subgroup analysis, participants from Asia demonstrated a higher frequency of hyperkalemia, as measured by both investigator reports [10% (Asia) vs. 7% (Europe/North America)] and laboratory assessments [19% (Asia) vs. 11% (Europe/North America)] [12].

The data regarding finerenone’s impact on eGFR in Asian patients present a somewhat conflicting picture. The meta-analysis by Xu *et al*. found that an eGFR decrease of ≥30% occurred more frequently in Asian patients receiving non-steroidal MRAs [2]. In contrast, the CONFIDENCE Asian subgroup analysis found that acute eGFR declines of >30% at day 30 were actually less frequent in Asia (3%) compared to Europe/North America (5%) [12]. The initial, modest decline in eGFR observed in some studies is a known hemodynamic effect of agents that reduce intraglomerular pressure, including MRAs and RAS inhibitors. This acute dip is typically not indicative of intrinsic kidney injury and often portends long-term renal preservation.

In summary, these data provide supportive evidence to support the idea that finerenone may work better in Asian populations. But it is also important to note that in subgroup analyses of large RCTs, no statistically significant differences in efficacy and safety were observed across ethnic groups. The underlying biological and environmental factors may help explain these different findings. As noted above, these are post-hoc subgroup observations, and formal statistical tests for treatment-by-ethnicity interaction were not significant for most endpoints; the apparent ethnic difference should therefore be regarded as hypothesis-generating rather than conclusive.

### POTENTIAL MECHANISMS FOR FINERENONE’S RESPONSE IN ASIAN POPULATIONS

Finerenone seems to work better in Asian patients, but this finding is not consistent across all studies. Even so, it makes us want to find the possible reasons behind it. We can find reasonable explanations in a mix of factors. These include genetic traits, common dietary habits, and unique baseline clinical features. All of these are more common in Asian patients with DKD.

### Genetic predisposition: the role of the *NR3C2* gene

The pharmacological target of finerenone is the mineralocorticoid receptor (MR), which is encoded by the *NR3C2* gene. This receptor plays a pivotal role in regulating sodium retention, fluid homeostasis, inflammation, and fibrosis [13]. There is growing evidence linking genetic variations in *NR3C2* to salt sensitivity, a trait known to be common in many Asian populations. Findings from a population-based dietary intervention study conducted in northern China, are particularly relevant. This study demonstrated a significant association between specific *NR3C2* gene polymorphisms (such as single nucleotide polymorphism rs3910053) and both the salt sensitivity of blood pressure and the long-term incidence of hypertension in Chinese adults [13].

This genetic link provides a clear biological hypothesis: a higher prevalence of specific *NR3C2* gene variants within Asian populations could lead to greater intrinsic MR pathway activity or heightened sensitivity to stimuli like dietary salt. This elevated baseline activity would make the MR pathway a more impactful target for pharmacological blockade with finerenone. Consequently, this could explain the more pronounced reductions in UACR and blood pressure observed in some studies of Asian patients.

### Dietary factors: the impact of high salt intake

Dietary patterns prevalent in many Asian countries may create a physiological environment where finerenone’s effects are amplified. High salt intake is a well-documented and common dietary pattern across Asia [1, 2].

The physiological link is direct: excessive salt intake can trigger renal MR activation, even independently of aldosterone levels. This activation promotes sodium retention, increases blood pressure, and drives pro-inflammatory and pro-fibrotic signaling in the kidneys [2, 13]. This creates a “two-hit” scenario in genetically susceptible individuals: a higher prevalence of salt-sensitive *NR3C2* polymorphisms (the first hit) combined with a diet that chronically activates the MR pathway (the second hit), making pharmacological MR blockade with finerenone a particularly high-impact intervention. It is plausible that chronic, diet-induced MR overactivation in many Asian patients creates a state of heightened cardiorenal stress where the anti-inflammatory, anti-fibrotic, and hemodynamic effects of an MRA like finerenone are magnified, leading to a greater therapeutic response.

### Pharmacokinetic considerations

Available pharmacokinetic (PK) data provide some reassurance that differential drug exposure is unlikely to fully explain the apparent ethnic difference in efficacy. A population pharmacokinetic/pharmacodynamic analysis of the Mineralocorticoid Receptor Antagonist Tolerability Study–Diabetic Nephropathy (ARTS-DN) and ARTS-DN Japan phase IIb studies found no significant ethnic differences in the PK/Pharmacodynamics (PD) relationships for UACR, serum potassium concentration, or eGFR; body weight, a potential driver of PK differences, was identified as a significant covariate only for volume of distribution and not for clearance [14]. Consistently, a recent review concluded that available pharmacokinetic data do not support clinically meaningful ethnicity-based differences in finerenone exposure that would justify separate dosing rules [15]. Nonetheless, dedicated, adequately powered pharmacokinetic studies specifically designed to compare Asian and non-Asian populations remain lacking; this is an important evidence gap, since undetected differences in drug exposure could in principle contribute to some of the observed differences in efficacy and hyperkalemia risk.

### Baseline clinical phenotypes

An important consideration when interpreting the observed differences in UACR response is the substantially higher baseline albuminuria in Asian cohorts relative to the global trial populations, patients with greater baseline albuminuria inherently have more capacity for absolute UACR reduction, without necessarily reflecting a superior pharmacodynamic response. Differences in the clinical characteristics of patients at the start of treatment may also contribute to the observed effects. Compared to global FIDELIO-DKD population [5], the Chinese participants in FIDELIO-DKD were younger (mean age 60.3 vs. 65.6 years), had a shorter duration of T2D (13.7 vs. 16.6 years). Despite that, the Chinese subgroup presented with a higher median baseline UACR (1,270 mg/g) than the overall study population (ranged from 833 to 867 mg/g) [7]. The same pattern appeared in FIGARO-DKD Chinese subgroup (median baseline UACR of Chinese and global populations were 742.12 and 308.18 mg/g, respectively) and CONFIDENCE Asian subgroup (Table 1). In the CONFIDENCE Asian subgroup, participants were younger (mean age 64 vs. 69 years), had lower body weight (70.4 vs. 91.4 kg), and body mass index (BMI) (26.2 vs. 31.8 kg/m²), and had higher median baseline UACR (663 vs. 504 mg/g) compared with the Europe/North America cohort [8, 12]. This higher baseline UACR represents an important confounder, as patients with greater baseline albuminuria inherently have more capacity for absolute reduction, which may contribute to the observed differences in relative UACR reductions between Asian and non-Asian populations.

Also, the placebo group in the FIDELIO-DKD Chinese subgroup had a higher event rate. The primary kidney composite endpoint occurred in 33.2% of Chinese patients on placebo, compared to 21.1% in the global population [7]. This tells us Chinese patients may have had a more severe or higher-risk form of DKD, means treatment can bring greater absolute benefits, which leads to a much higher RRR.

While a higher baseline UACR does not prove enhanced biological efficacy, it provides a greater absolute range for therapeutic reduction. This may partially explain why the relative UACR reductions appear larger in Asian cohorts in some analyses, as a larger absolute drop from a higher starting point yields a more pronounced percentage change. Of course, efficacy must always be balanced with a careful assessment of the safety profile.

Sex distribution also differs between Asian and non-Asian trial participants: males predominate among Asian subgroups to a greater extent than among non-Asian participants. A FIDELITY post-hoc analysis by age and sex found that the cardiovascular and kidney benefits of finerenone were generally consistent across sex categories (interaction *P* = 0.99 for the cardiovascular composite and = 0.85 for kidney outcomes), although the reduction in hospitalization for heart failure appeared more pronounced in males (interaction *P* = 0.02) [16]. Because existing Asian and Chinese subgroup analyses have not been formally adjusted for this sex imbalance, sex may be a partial, unadjusted contributor to the apparent differences in treatment response between Asian and non-Asian populations.

### Potential causes for increased hyperkalemia

Several hypotheses have been proposed to explain the higher reported rates of hyperkalemia in Asian cohorts. (1) Concomitant medications: The CONFIDENCE trial analysis noted that the concomitant use of diuretics was substantially lower in the Asian cohort (21%) compared to the Europe/North America cohort (49%). The authors suggested this difference may have contributed to the higher hyperkalemia rate, as diuretics can promote potassium excretion [12]. (2) Body weight: the Xu *et al*. meta-analysis posited that the lower average body weight characteristic of Asian patients could lead to a relatively higher drug dose per kilogram of body mass, potentially increasing the risk of dose-dependent side effects like hyperkalemia [2]. (3) Reporting differences: the FIGARO-DKD China analysis raised the possibility of “over-reporting.” It was suggested that the serum potassium threshold used by investigators in China to report hyperkalemia as an adverse event may have been lower, based on local guidelines, than in other regions [8].

It is critical, therefore, for the clinician to distinguish between statistical incidence and clinical impact. While laboratory-defined hyperkalemia was more frequent, events requiring intervention—such as hospitalization or treatment discontinuation—were rare and comparable to non-Asian populations. This suggests the risk is manageable but requires a structured and vigilant monitoring protocol rather than avoidance of the therapy [8, 12].

### CLINICAL IMPLICATIONS AND CONCLUSIONS

To date, Asia still faces a monumental public health challenge in managing DKD. The new published “Implementation of Global CKD Guidelines in Asia” [1] indicated that underutilization of newer, effective pharmacotherapies, especially the nsMRA finerenone, is one of the primary concerns. Delayed DKD diagnosis due to low awareness and screening rates, prescribing restrictions in countries like Japan and Singapore [finerenone is positioned as a second-line therapy with prior Sodium-Glucose Co-transporter 2 inhibitor (SGLT2 inhibitor) therapy], serum potassium monitoring requirements or lack of monitoring protocols, cost and access, are the multiple barriers to finerenone implementation.

Addressing these gaps will be essential for refining clinical guidelines and optimizing the use of this important therapy in the high-risk Asian DKD population. First, for all patients with T2D, regular screening for albuminuria using the UACR is imperative; this screening helps identify patients early who could benefit from cardiorenal protective therapies like finerenone, and it is especially critical because trial data show many Asian patients have high baseline albuminuria levels, which means they have significant underlying risk [1, 8, 12]. Second, early combination therapy strategy like simultaneous addition of finerenone and SGLT-2i is expected to transform clinical practice patterns. The traditional stepwise “add-on” approach (finerenone with prior SGLT-2i) may lead to therapeutic inertia and irreversible disease progression, this more proactive strategy which supported by CONFIDENCE study is likely to result in earlier disease control and improved long-term outcomes, particularly in high-risk patients [11, 17].

Given the consistent signal of increased hyperkalemia incidence in Asian cohort, a structured monitoring schedule is non-negotiable. Clinicians could adopt the protocol suggested by the APSN guidelines [3], which involves checking serum potassium at baseline, within 1–2 weeks of initiation or dose titration, and regularly thereafter, adjusting management based on established thresholds.

Regarding the FINE-ARTS HF trial (finerenone in heart failure with mildly reduced or preserved ejection fraction), detailed subgroup analyses by ethnicity, including data specifically on Asian patients, have not yet been reported in the published literature at the time of writing; we were therefore unable to incorporate FINE-ARTS HF-specific Asian subgroup data into this review. As a partial substitute, the earlier ARTS-HF Japan phase IIb study provides supportive, albeit preliminary, evidence that finerenone was well tolerated in Japanese patients with worsening heart failure and diabetes and/or chronic kidney disease, with a low overall incidence of hyperkalemia (2.8%) across dose groups [18]. This is a different trial from FINE-ARTS HF, and any comparison should be made with caution.

To place these findings within a broader therapeutic context, differential efficacy by ethnicity has also been reported for other nephroprotective drug classes. A systematic review and meta-analysis of randomized controlled trials found that the glucose-lowering efficacy of SGLT2 inhibitors, and to a lesser extent DPP-4 (Dipeptidyl Peptidase-4) inhibitors, was greater in studies of predominantly Asian ethnicity compared with studies of predominantly White ethnicity, whereas no such ethnic difference was observed for GLP-1 (Glucagon-Like Peptide-1) receptor agonists [19]. This suggests that the apparently greater efficacy observed in Asian patients may not be unique to finerenone, but could instead reflect a broader pattern of response to certain nephroprotective and cardiometabolic interventions in Asian populations with DKD.

In conclusion, finerenone has been firmly established as a cornerstone therapy for reducing the risk of kidney and cardiovascular events in patients with DKD. While evidence from major analyses of pivotal trials shows consistent efficacy across diverse global populations, compelling data from a meta-analysis and a key trial’s subgroup analysis suggest a potentially enhanced benefit on both renal outcomes and critical surrogate markers in Asian patients. This intriguing possibility of greater efficacy is supported by plausible underlying mechanisms, including genetic factors related to the mineralocorticoid receptor pathway (*NR3C2* gene) and high-salt dietary habits that are prevalent in many parts of Asia. As early screening awareness and actions improving in Asian, careful and individualized approach to DKD treatment would help clinicians confidently maximize the significant cardiorenal benefits of finerenone while ensuring patient safety. It should be emphasized that these conclusions rest primarily on post-hoc subgroup analyses with largely non-significant formal interaction tests, modest sample sizes in some subgroups, and incomplete pharmacokinetic characterization in Asian populations; dedicated prospective studies are needed to confirm whether the apparent enhanced efficacy in Asian patients is real and clinically actionable.

## Data Availability

The data underlying this article are available in the article.
